# Macular Hemorrhage as the First Manifestation of Leukemia

**DOI:** 10.3390/diagnostics16101518

**Published:** 2026-05-16

**Authors:** Bogumiła Wójcik-Niklewska, Natalia Kwasniewska, Adrian Smędowski

**Affiliations:** 1 Department of Pediatric Ophthalmology, Faculty of Medical Sciences in Katowice, Medical University of Silesia, 40-514 Katowice, Poland; 2 Professor Kornel Gibiński University Hospital Center, Medical University of Silesia, 40-514 Katowice, Poland; 3 Students’ Scientific Society, Department of Ophthalmology, Faculty of Medical Sciences in Katowice, Medical University of Silesia, 40-752 Katowice, Poland; 4 GlaucoTech Co., Ltd., 40-282 Katowice, Poland

**Keywords:** acute lymphoblastic leukemia, macular hemorrhage, leukocytosis, thrombocytopenia

## Abstract

Acute lymphoblastic leukemia (ALL) is a malignant neoplasm of the blood and bone marrow characterized by the uncontrolled proliferation of precursor cells of B- or T-lymphocyte lineage. Usually, the disease arises because of spontaneous mutations in bone marrow cells. Risk factors include genetic predisposition, exposure to ionizing radiation, prior chemotherapy or radiotherapy, and certain environmental factors. Clinical manifestations may include recurrent infections, anemia, and an increased tendency toward bleeding and stroke. A 12-year-old boy presented to the emergency department with a sudden decrease in visual acuity in the right eye. Best-corrected visual acuity (BCVA) in the right eye was 0.02, and intraocular pressure (IOP) was 16 mmHg. Ophthalmologic examination revealed a macular hemorrhage in the right eye. Blood samples were obtained for laboratory analysis. Complete blood count demonstrated leukocytosis with a white blood cell (WBC) count of 362.58 × 10^3^/µL, thrombocytopenia with a platelet (PLT) count of 87 × 10^3^/µL, hemoglobin (Hgb) level of 8.7 g/dL, and a red blood cell (RBC) count of 3.46 × 10^6^/µL. The patient was subsequently referred to the Department of Pediatric Hematology, where the diagnosis of acute lymphoblastic leukemia of B-cell precursor origin was confirmed. Appropriate systemic therapy targeting the underlying disease was initiated.

**Figure 1:**
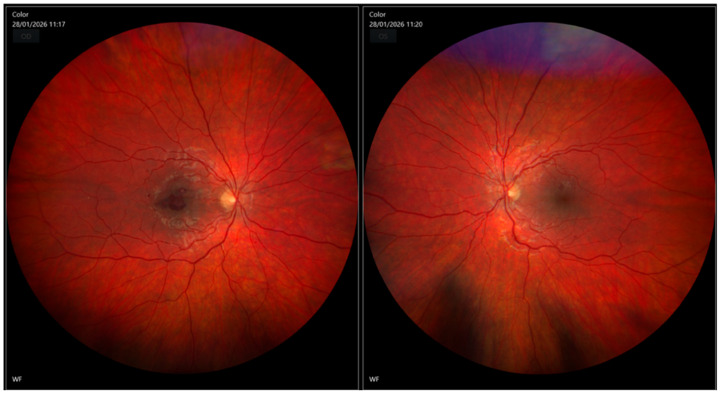
Fundus photography of both eyes revealed pathological changes in the macular region of the right eye. In the area of the fovea, an irregular, dark-red lesion corresponding to a preretinal hemorrhage was observed. The optic discs exhibited well-defined margins, with no signs of edema. Retinal vessels showed no significant morphological abnormalities. Other features characteristic for leukemia like Roth spots, venous tortuosity, cotton wool spots, optic disc edema, serous retinal detachment were not present. The observed hemorrhagic lesion was confined to the right eye. In the left eye the optic disc appeared normal with sharp margins and there was no evidence of hemorrhages or other pathological changes. The overall presentation is consistent with a macular hemorrhage correlated with a sudden decrease in visual acuity. Similar findings in pediatric patients with leukemia are reported extremely rarely. Tan BH et al. described a case of a child with B-cell precursor acute lymphoblastic leukemia who presented with an extensive premacular subhyaloid hemorrhage and multiple Roth spots on fundus examination. Conservative management combined with systemic chemotherapy resulted in complete recovery of visual acuity after five months [1]. In a case report by Dampuru et al., an 11-year-old girl presented with visual disturbances, subconjunctival hemorrhages, multiple preretinal hemorrhages, and vitreous hemorrhage, which prompted further systemic evaluation and led to the diagnosis of acute leukemia [2]. Carter et al. reported retinal hemorrhages and retinal detachment as manifestations of ALL relapse [3]. Complete blood count in this patient demonstrated leukocytosis with a white blood cell (WBC) count of 362.58 × 10^3^/µL, thrombocytopenia with a platelet (PLT) count of 87 × 10^3^/µL, hemoglobin (Hgb) level of 8.7 g/dL, and a red blood cell (RBC) count of 3.46 × 10^6^/µL; hematocrit (HCT) was decreased at 27.3%. Differential count demonstrated a predominance of lymphocytes (85%). Red cell distribution width (RDW) was elevated at 21.6% and RDW-SD (red cell distribution width—standard deviation) was also increased at 56.4 fL, indicating significant anisocytosis. Coagulation analysis showed an activated partial thromboplastin time (APTT) within normal limits. There was no history of trauma, and other causes of unilateral macular hemorrhage like Valsalva maneuver, ocular rubbing, and high blood pressure were ruled out. Additionally, the patient reported recent generalized weakness and reduced physical performance.

**Figure 2:**
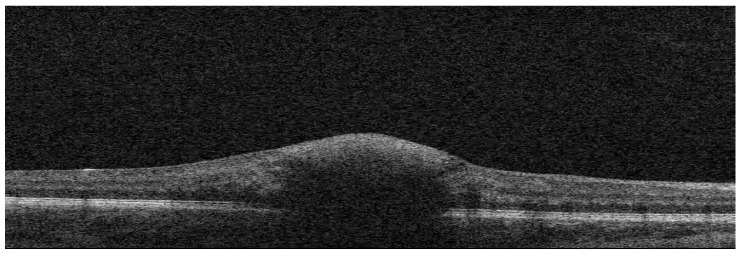
Optical coherence tomography (OCT) of the macula in the right eye demonstrates the presence of hyperreflective material within the foveal region. The dome-shaped configuration of the lesion is more consistent with a sub-internal limiting membrane (sub-ILM) hemorrhage. Disruption of the normal retinal layer architecture is evident in the central area, with localized thickening and shallowing of the foveal depression. In this patient, management was focused on the treatment of the underlying systemic disease. Macular hemorrhage resolved and BCVA in the right eye improved to 0.7. Leukemia is the most common malignancy of childhood, accounting for nearly 25% of all cancers diagnosed in individuals under 20 years of age [4]. The pathophysiology of fundus changes in patients with leukemia is multifactorial. Retinal and macular hemorrhages may result from severe thrombocytopenia, anemia, leukostasis, or increased blood viscosity. Additionally, leukemic infiltration of retinal vessel walls leads to their weakening and increased permeability, predisposing them to hemorrhagic extravasation [5,6]. Macular hemorrhage, particularly when unilateral, requires careful differential diagnosis. Potential alternative causes include Valsalva retinopathy, ocular rubbing, minor ocular or head trauma, and systemic hypertension [4,7,8]. These conditions should be considered, especially in pediatric patients presenting with acute visual symptoms, to avoid misattribution of findings solely to hematologic disease. In the present case, the absence of trauma history, normal blood pressure, and accompanying systemic abnormalities supported a hematologic etiology. Valsalva maculopathy is a clinical condition presenting with sudden, painless visual impairment or a central scotoma. It results from the rupture of superficial retinal capillaries caused by a rapid rise in intraocular venous pressure, typically secondary to increased intrathoracic or intra-abdominal pressure [9]. Macular hemorrhages are uncommon in cases of accidental trauma and, when they do occur, they are typically unilateral, limited in number, and confined to the posterior pole [10]. Beyond hemorrhagic findings, ophthalmic manifestations of leukemia may include Roth spots, venous tortuosity, cotton wool spots, optic disc edema, and, less commonly, serous retinal detachment or direct leukemic infiltration [5,6]. These features were not observed in our patient but their presence in other cases underscores the importance of comprehensive fundoscopic evaluation. Tan et al. described extensive premacular subhyaloid hemorrhage accompanied by multiple Roth spots, suggesting a more diffuse retinal involvement [1]. Dampuru et al. reported widespread hemorrhagic manifestations, including vitreous hemorrhage, indicating advanced ocular pathology at presentation [2]. The more localized hemorrhage in our case may reflect an earlier stage of ocular involvement or a different underlying pathophysiological mechanism like a localized vascular fragility rather than widespread leukemic infiltration. This case underscores the important role of the ophthalmologist in the early detection of systemic diseases. Sudden, unilateral deterioration of visual acuity due to macular hemorrhage in a child should prompt extended diagnostic evaluation, including consideration of neoplastic conditions. It is essential to perform complete blood count with differential and assess coagulation parameters, as leukemias are the most common malignancies in children. Early diagnosis of acute lymphoblastic leukemia is crucial for patient prognosis and the timely initiation of appropriate targeted therapy. Ocular manifestations may represent the first sign of a life-threatening disease.

## Data Availability

The data presented in this study are available on request from the corresponding author due to privacy.

## Abbreviations

The following abbreviations are used in this manuscript:

ALL	Acute lymphoblastic leukemia
BCVA	Best-corrected visual acuity
IOP	Intraocular pressure
CBC	Complete blood count
WBC	White blood cells
RBC	Red blood cells
PLT	Platelets
RDW	Red cell distribution width
RDW-SD	Red cell distribution width—Standard deviation
APTT	Activated partial thromboplastin time
OCT	Optical coherence tomography
